# Critical insights on “Association of the C allele of rs479200 in the EGLN1 gene with COVID‑19 severity in Indian population: a novel finding”

**DOI:** 10.1186/s40246-024-00618-4

**Published:** 2024-05-24

**Authors:** Nimita Deora, Priya Agrohi, Prashant K Mallick, Abhinav Sinha

**Affiliations:** 1 Department of Epidemiology, ICMR-National Institute of Malaria Research, New Delhi, 110077 India; 2 Department of Molecular Biology, ICMR-National Institute of Malaria Research, New Delhi, 110077 India; 3 Academy of Scientific and Innovative Research (AcSIR), Ghaziabad, India

**Keywords:** EGLN1, C-allele, COVID-19, Severity

## Abstract

The recent article by Harit et al. in Human Genomics reported a novel association of the C allele of rs479200 in the human EGLN1 gene with severe COVID-19 in Indian patients. The gene in context is an oxygen-sensor gene whose T allele has been reported to contribute to the inability to cope with hypoxia due to increased expression of the EGLN1 gene and therefore persons with TT genotype of EGLN1 rs479200 are more susceptible to severe manifestations of hypoxia. In contrast to this dogma, Harit et al. showed that the C allele is associated with the worsening of COVID-19 hypoxia without suggesting or even discussing the scientific plausibility of the association. The article also suffers from certain epidemiological, statistical, and mathematical issues that need to be critically elaborated and discussed. In this context, the findings of Harit et al. may be re-evaluated.

We read with high intrigue the ‘novel’ findings published by Harit et al. in Human Genomics that attempted to demonstrate that the C allele of rs479200 in the EGLN1 gene has statistically significant higher odds of being associated with COVID-19 severity in an Indian cohort [1]. However, the article by Harit et al. appears to be unclear, confusing, and at certain places, scientifically incorrect to conclude that the claims made by the authors are justified.

As per our conceptual understanding, the oxygen-responsive egg-laying defective nine homolog of *C. elegans* in humans or the EGLN1 gene produces prolyl hydroxylases responsible for proteasomal degradation of the α-subunits of the hypoxia-inducible factor (HIF-α) via hydroxylation and polyubiquitination [2, 3]. Under hypoxia, EGLN1 is inhibited leading to the accumulation of HIF-α and the formation of functional transcriptional factor through its heterodimerization with HIF-β [3, 4]. This HIF-αβ complex stimulates the transcription of a cascade of genes responsible for adaptive responses to hypoxia, collectively termed hypoxia-responsive elements or HREs, that tend to mitigate the negative effects of hypoxia and thus prevent the severity of hypoxia. These downstream compensatory effects include restoring oxygen supply (angiogenesis, erythrocytosis, ventilation), reducing oxygen consumption, augmenting hypoxia tolerance, and redox pH homeostasis, to name a few [2, 5].

It has been documented that the rs479200 TT genotype of EGLN1 is associated with significantly higher expression of EGLN1 as compared to the CT and CC genotypes [6, 7]. Under normoxia, the individuals with rs479200 TT genotypes are phenotypically indifferent to those with CC and CT genotypes, with respect to the adaptive responsiveness to hypoxia. However, this surplus EGLN1 (rs479200 TT genotype) under hypoxic stress has been implicated in poor adaptive responses to hypoxia thus manifesting in severe hypoxia akin to the high altitude pulmonary edema (HAPE) as observed in Indian and Peruvian populations [6, 8]. Our concerns for the conclusions derived by Harit et al. are as follows:

1. In this context, the association of the rs479200 C allele of EGLN1 with COVID-19 severity as claimed by Harit et al. [1] appears to be scientifically unexplainable and the plausibility of the same has not been discussed by Harit et al. or discussed only in the context of the T allele of rs479200. The T allele of rs479200 has a higher scientific plausibility of being associated with the worsening of hypoxia as it happens in COVID-19 severe patients as compared to the C allele and therefore the novelty claimed by the authors is unclear and left unexplained. The authors postulated a hypothesis on how the C allele would be associated with COVID-19 severity but explained the contrary in the discussion section. We were also unable to find any appropriate explanation for why they had witnessed utterly different ‘novel’ outcomes. Moreover, the association between rs479200 genotypes and EGLN1 expression are tissue-dependent. Exploratory eQTL analyses of the rs479200 genotype-expression association data from the gene tissue expression (GTEx) portal (https://gtexportal.org/home/locusBrowserPage/EGLN1) showed statistically significant association (*P* < 0.00015) between the rs479200 genotypes and EGLN1 expression in only two tissues (adrenal gland and brain-nucleus accumbens in basal ganglia). More importantly, both tissues had strikingly contrast difference in the association wherein the brain showed higher EGLN1 expression with TT genotype as opposed to the adrenal gland which showed higher gene expression with the CC genotype. This should have been discussed by Harit et al. as they and the previously published literature used blood [peripheral blood [1, 6] and venous blood [7]] for expression analysis whereas the GTEx data shows no genotype-expression association in EGLN1 rs479200 for whole blood sample.
2. It is unclear how the authors recruited and labelled the patients as ‘asymptomatic’ and whether they followed them to see the progression of COVID-19 infection to the need for supplemental oxygen and/or severity. It is also unclear why the authors used an indirect criterion (type of supplemental oxygen delivery system) to categorize COVID-19 patients rather than more direct hypoxia indices like SpO2. It is exceedingly unlikely that the authors got basic demographic information (age, gender, type of oxygen device) but did not obtain the key parameter, SpO2, from the enrolled COVID-19 patients. Although they stated that they did not obtain detailed physiological parameters, they did receive critical parameters of patients from the ICUs. This would have affected the classification of cases and thus the analyses. The investigators’ classification of COVID-19 patients as mild (needed oxygen through a low-flow system) and severe (needed oxygen through a high-flow system or ventilator) appears to be arbitrary as the use of degree of flow of oxygen is not a direct criterion for determining COVID-19 associated severity. Instead, SpO2 level might have been used to classify the disease severity (Mild: no dyspnea; Moderate: SpO2 ≥ 94%; Severe: SpO2 < 94%) as a direct measure of hypoxia [9]. In addition, the possibility of progression of ‘mild’ COVID-19 to severe disease was also not monitored (as with the possibilities of progression from asymptomatic to mild) which also might have affected the results. Further, the reference cited by the authors for using their criteria for classifying COVID-19 severity [10] also does not appear to mention the criterion used by Harit et al.
3. Based on the above classification of severity, the authors concluded their result concerning the severity of COVID-19 patients which may seem misleading given the manuscript’s main title. The study authors should have avoided generalizing the term “severe” and instead concluded their findings based on the need and degree of oxygen flow. Moreover, the confounding effects of other comorbidities and the recent findings regarding novel functions of such or similar genes need to be discussed in more detail to provide a complete picture [11, 12]. It is also unclear how authors arrived at such strong conclusions when the similarities between the pathophysiology of severe COVID and HAPE are debatable. The same was recognized by the authors but not mentioned as a serious limitation of the conclusion.
4. The manuscript also suffers from some mathematical and statistical errors as evident from the Table 1 and supplementary Tables 1 and 2 of Harit et al. [1]. The overall sample size mentioned in the manuscript (*N* = 158) does not align with the sample size provided in the supplementary Table S2 (*N* = 162 by combining males and females and *N* = 167 by combining clinical categories which are asymptomatic, mild, and severe COVID-19 for rs479200). The number of males and females also varies between supplementary Table S1 (*n* = 158 but reported as 167; 120 males and 38 females) and supplementary Table S2 (*n* = 162; 123 males and 39 females) and so does the number of severe patients (58 in S1 but reported as 67 and 67 in S2). The totalling of different genotypes of rs479200 by gender (*n* = 162) and COVID-19 clinical categories (*n* = 167) also does not match in S2 and the explanation for the same has been omitted. Additionally, in the additive model for odds ratio calculations in Table 1, the odds ratios mentioned do not match with the ratios calculated by us based on the data from supplementary Table S2. Since the authors claim the conclusion from the statistically significant association based on the additive model, as mentioned in the abstract, this oversight causes the inheritance model to be analyzed and interpreted incorrectly, which would distort the study’s findings. To add to the mistakes, the adjusted and unadjusted odds ratios are wrongly mentioned in the abstract. To accurately evaluate the results, these points need to be clarified as such errors have substantial implications for the interpretation of the study’s findings. Discrepancies in the reported sample size compromise the validity of statistical analyses and even if we consider the sample size given in Table S2 to be correct, the inaccuracies in the odds ratio could still result in misinterpretation of the strength of associations between the C allele of rs479200 and severe category of COVID-19.
5. Further, the authors’ observation of the rs479200 C allele in a frequency of 0.66 in the severe group is contradictory to the observation of the rs479200 C allele in a frequency of 0.37 among 1022 healthy Indians studied in various ethnic groups across India [13]. However, such a high frequency (≈ 0.71) of the C allele of rs479200 was reported in populations belonging to high altitudes [6, 8]. It appears that the severe COVID-19 patients in Harit et al. may belong to a limited ethnic group belonging to a high-altitude area which may then explain the reported high frequency of C allele of rs479200. Whereas the asymptomatic and mild COVID-19 groups may have accommodated samples from various ethnic groups that provide a different allele frequency. In addition, these groups still do not match the frequency reported in the 1000 genome database or the IndiGenomes SNP database reported for healthy individuals worldwide. Moreover, the IndiGenomes database also showed that this locus (rs479200) deviates from the Hardy-Weinberg equilibrium. Thus, it appears that this study might have added a group of healthy population to estimate the baseline frequency of the rs479200 alleles.

Therefore, in light of the above considerations, the findings presented by Harit et al. as novel and clear documentation of a scientific hypothesis are likely to be fallible and need a strong second thought.

## Data Availability

No datasets were generated during the current study.

## Abbreviations

COVID-19: (Corona Virus Disease 2019)
eQTL: (Expression Quantitative Trait Loci)
EGLN1: (Egg Laying Nine gene 1)
GTEx: (Gene Tissue Expression)
HAPE: (High Altitude Pulmonary Edema)
HIF: (Hypoxia Inducing Factor)
HRE: (Hypoxia Responsive Elements)
pH: (potential of hydrogen)
SpO_2_: (Peripheral oxygen saturation)
